# CLINICIANS’ PERSPECTIVES REGARDING THE INFLUENCE OF ADVERSE SOCIAL DETERMINANTS ON ACUTE CARE RESOURCE USE

**DOI:** 10.1093/geroni/igae098.3208

**Published:** 2024-12-31

**Authors:** Onome Osokpo, Karen Hirschman, Elizabeth Shaid, Kathleen McCauley, Mary Naylor

**Affiliations:** University of Illinois Chicago, Chicago, Illinois, United States; University of Pennsylvania, Philadelphia, Pennsylvania, United States; Penn Nursing, Merion Station, Pennsylvania, United States; University of PA School of Nursing, Ardmore, Pennsylvania, United States; University of Pennsylvania, Philadelphia, Pennsylvania, United States

## Abstract

Adverse Social Determinants of Health (SDoH) factors place older adults living with multiple chronic conditions at risk for poor outcomes during transitions from hospital to home. The Transitional Care Model (TCM) intervention, an advanced practice registered nurse (APRN)–led, team-based care-management strategy, improves outcomes throughout these transitions. However, little is known about the SDoH factors identified by APRNs delivering the TCM that may influence acute-care resource use. Guided by the Healthy People 2030 SDoH framework, we present the results of a descriptive qualitative analysis of 36 quality improvement and support meetings with clinical team members (i.e., APRNs, clinical coordinators) at three healthcare systems (7 hospitals/4 states) responsible for the delivery of the TCM to older adults hospitalized with heart failure, COPD, or pneumonia as part of the MIRROR-TCM trial. A review of clinical team meetings from January-June 2022, where teams met and reviewed and completed root cause analyses related to TCM care delivery challenges and acute-care resource use, revealed that most of the SDoH factors occurred within the Healthcare Access and Quality and Social and Community Context domains of Healthy People 2030 SDoH framework. These challenges include barriers to scheduling appointments, communication lapses amongst providers, reluctance or refusal by patients and/or families for home visits, and language barriers with non-English speakers. Strategies to address these challenges include, advocating for and coordinating follow-up appointments, use of telehealth, and healthcare-community partnerships to address patients’ concerns and unmet health-related social needs. Engaging these transitional care strategies may result in improved posthospitalization outcomes.

